# Identification of single nucleotide polymorphisms in bovine *CARD15 *and their associations with health and production traits in Canadian Holsteins

**DOI:** 10.1186/1471-2164-8-421

**Published:** 2007-11-15

**Authors:** Sameer D Pant, Flavio S Schenkel, Ivan Leyva-Baca, Bhawani S Sharma, Niel A Karrow

**Affiliations:** 1 Centre for Genetic Improvement of Livestock, Department of Animal and Poultry Science, University of Guelph, Guelph, Ontario, N1G 2W1, Canada

## Abstract

**Background:**

Toll-like receptor-2 (TLR2) and Caspase Recruitment Domain 15 (CARD15) are important pattern recognition receptors that play a role in the initiation of the inflammatory and subsequent immune response. They have been previously identified as susceptibility loci for inflammatory bowel diseases in humans and are, therefore, suitable candidate genes for inflammatory disease resistance in cattle. The objective of this study was to identify single nucleotide polymorphisms (SNPs) in the bovine *TLR2 *and *CARD15 *and evaluate the association of these SNPs with health and production traits in a population of Canadian Holstein bulls.

**Results:**

A selective DNA pool was constructed based on the estimated breeding values (EBVs) for SCS. Gene segments were amplified from this pool in PCR reactions and the amplicons sequenced to reveal polymorphisms. A total of four SNPs, including one in intron 10 (c.2886-14A>G) and three in the exon 12 (c.3020A>T, c.4500A>C and c.4950C>T) were identified in *CARD15*; none were identified in *TLR2*. Canadian Holstein bulls (n = 338) were genotyped and haplotypes were reconstructed. Two SNPs, c.3020A>T and c.4500A>C, were associated with EBVs for health and production traits. The SNP, c.3020A>T, for example, was associated with SCS EBVs (p = 0.0097) with an allele substitution effect of 0.07 score. When compared to the most frequent haplotype Hap12(AC), Hap22(TC) was associated with increased milk (p < 0.0001) and protein (p = 0.0007) yield EBVs, and hap21(TA) was significantly associated with increased SCS EBV(p = 0.0120). All significant comparison-wise associations retained significance at 8% experimental-wise level by permutation test.

**Conclusion:**

This study indicates that SNP c.3020A>T might play a role in the host response against mastitis and further detailed studies are needed to understand its functional mechanisms.

## Background

Inflammatory diseases such as mastitis, inflammatory bowel disease, metritis and laminitis are economically important diseases for the dairy industry. Mastitis is the most common amongst them, alone accounting for losses in excess of $2 billion to the US dairy industry [1]. A number of therapeutic, prophylactic and management strategies have been proposed to minimize this complex disease. However, a widely accepted complimentary strategy is based on improving the host genetics through selective breeding. Since it is difficult to use a direct index to measure the mastitis phenotype, milk somatic cell score (SCS) is often used as an indirect index to select animals for breeding [2]. Milk SCS is a log_2 _score of the milk somatic cell count and has been genetically correlated with clinical mastitis (r = 0.7) [3]. It has been demonstrated in sheep that intramammary infections occur less frequently after one generation of breeding for low SCS[4]. Breeding strategies in dairy cattle are similarly based on selection for low estimated breeding values (EBVs) for SCS [5].

The etiology of mammary inflammatory disorders is diverse and may include infections caused by bacteria, parasites, viruses and fungi. In order for an effective host immune response to occur against a wide variety of pathogens, the host must possess receptors that recognize conserved molecular patterns associated with different classes of pathogens. These are referred to as pathogen associated molecular patterns (PAMPs). Receptors that recognize PAMPs are collectively referred to as pattern recognition molecules (PRMs) [6]. The PRMs are expressed widely by different cells including monocytes, granulocytes, dendritic cells and epithelial cells [7]. PAMP recognition leads to the secretion of cytokines and chemokines by the epithelial cells that recruit effector cells (neutrophils and monocytes) of the immune system to the site of infection where they contribute to the host inflammatory immune response and subsequent acquired immune response.

Phagosomal toll-like receptor-2 (TLR2) is involved in recognizing PAMPs associated with Gram-positive bacteria. Murine studies for example, have demonstrated that TLR2 is in involved in the early recruitment of neutrophils in response to intraperitoneal challenge with either *Staphylococcus aureus*, or peptidoglycan (PGN) from *S. aureus *[8]. Ruminant studies have demonstrated that TLR2 gene (designated as '*TLR2*') expression occurs in dermal and gut-associated tissues [9], and is highly induced during mastitis caused by *S. aureus *[10]. Bovine *TLR2 *was recently radiation hybrid mapped to *Bos taurus *autosome (Bta) 17 [11].

Caspase Recruitment Domain 15 (CARD15), also known as Nucleotide Oligomerization Domain 2 (NOD2), is a cytosolic protein capable of initiating inflammation following PAMP recognition. The gene encoding bovine CARD15 (designated as '*CARD15*') is located on Bta18. It was previously categorized as a member of the CATERPILLER family, but was reassigned to the phylogenetically conserved NLR (NACHT-LRR) protein family [12]. CARD15 shares a common tripartite domain structure with other members of this family. The tripartite domain consists of a carboxy (C)-terminal leucine rich repeat (LRR) domain; a central NACHT (NAIP CIITA HET-E and TP1) domain, and an amino (N) – terminal domain that is composed of two CARD domains. The LRR domain is involved primarily in the recognition of bacterial peptidoglycans (PGN), whereas the central NACHT domain facilitates self-oligomerization and has ATPase activity. The CARD domains are known to interact with CARD containing serine/threonine kinase Rip2 (known as RICK), via homophilic CARD-CARD interaction; this leads to the activation of NF-κB [13].

The major portion of the PGN recognition system in mammals is constituted by CARD15 along with NOD1 and TLR2 [14]. CARD15 is involved in intracellular recognition of muramyl dipeptide (MDP), the minimal bioactive structure of PGN, which is common to the cell wall of both Gram-positive and gram-negative bacteria. Therefore, CARD15 acts as a general sensor of bacterial infection [15,16]. A recent study suggests that NOD2 might be involved in sensing of PGN motifs of *S. aureus*, after its phagocytosis [17]. There are several reports of interactions between CARD15 and other PRMs, especially TLR2 [18-20]. This demonstrates the importance of CARD15 in signalling events associated with recognition of different PAMPs. Polymorphisms, particularly single nucleotide polymorphisms (SNPs), within the genes coding for different receptor proteins may impair the ability of certain individuals to respond properly to infections [21]. In the case of *TLR2 *and *CARD15*, they have been identified as susceptibility loci for different inflammatory bowel diseases in humans [22-25]. It is possible that SNPs within these genes influence other inflammatory diseases such as mastitis in cattle. Therefore, understanding the genetic variation underlying PRMs might help livestock breeders to identify and select animals with enhanced resistance to mastitis for breeding programs. The purpose of this study was to identify SNPs in the bovine *TLR2 *and *CARD15 *and to evaluate the association of these SNPs with SCS and other production traits in a population of Canadian Holstein bulls.

## Results

### SNP Detection

**Figure 1:**
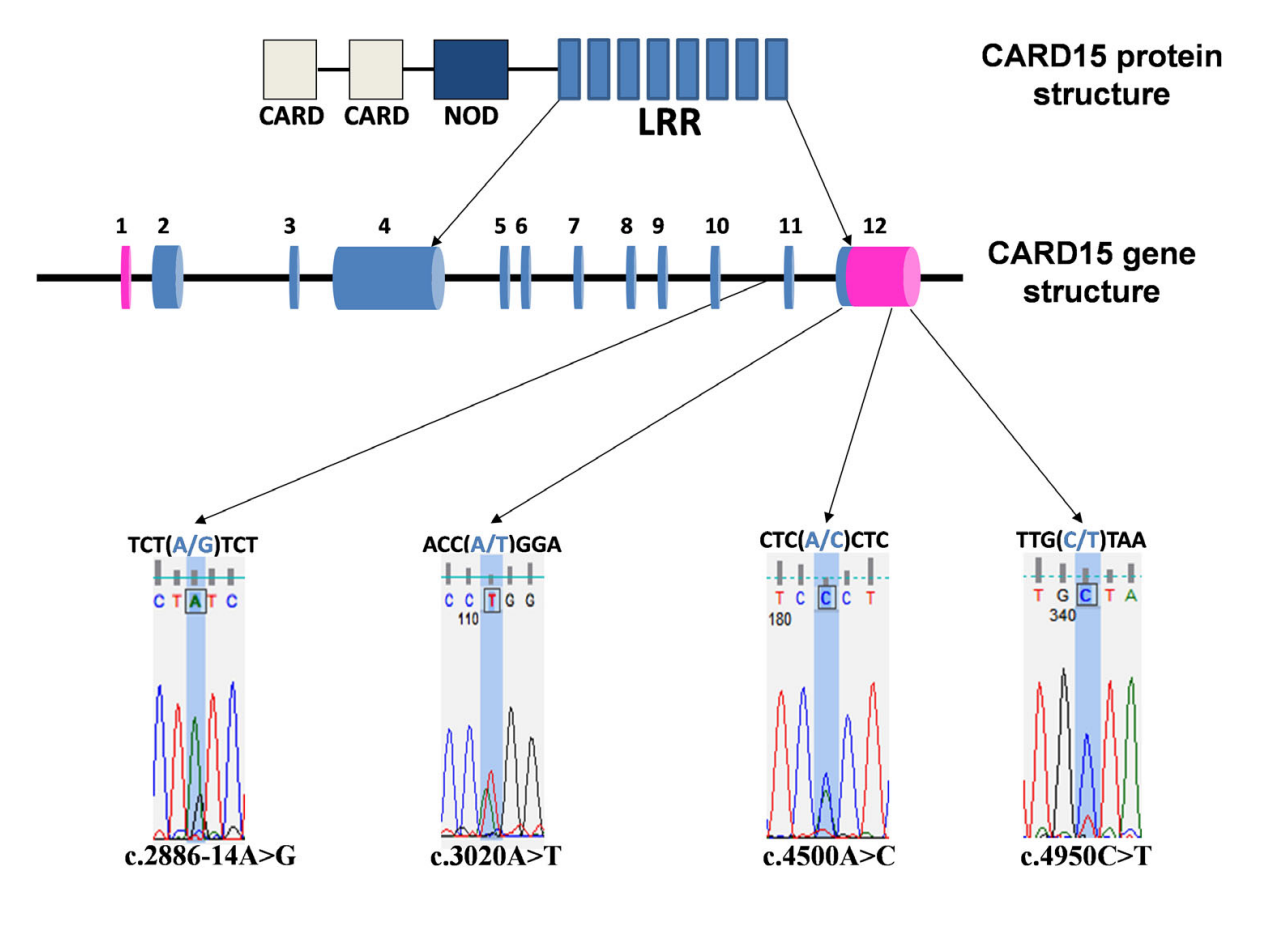
***CARD15 *structure and location of SNPs**. *CARD15 *structure showing the exons coding the LRR domain of the protein and location of identified SNPs.

Investigation of the coding exon, flanking introns and promoter sequences of *TLR2 *revealed no polymorphisms. Investigation of exonic, flanking intronic and promoter sequences of *CARD15 *revealed the presence of four SNPs, including two transitions: A ↔ G at position c.2886-14A>G and A ↔ T at position c.3020A>T, and two transversions: A ↔ C at position c.4500A>C and C ↔ T at position c.4950C>T (Figure 1). The nomenclature adopted for the SNPs was based on the convention described by the human genome variation society [26]. No SNPs were found in the promoter sequence from the set of animals used in this study. SNP c.2886-14A>G was the only polymorphism found in the flanking intronic sequence (14 base pairs upstream of exon 11). All the other SNPs were found in exon 12. SNP c.3020A>T was found in the coding sequence of exon 12 and is non-synonymous; allele 'A' producing leucine and allele 'T' producing glutamine in the peptide sequence. The SNPs were submitted to the National Centre for Biotechnology Information (RefSNP rs43710287, rs43710288, rs43710289, rs43710290) and were released in dbSNP build 126.

### Genotypic and allelic frequencies

The genotypic and allelic frequencies are summarized in Table 1. The individual frequencies of the genotypes were in Hardy-Weinberg equilibrium for all the SNPs, as determined by Chi-square test. The calculated Chi-square values ranged from 0.06 to 1.76 and were all non-significant (p < 0.05). The linkage disequilibrium was evaluated for all pairs of SNPs using r^2^. The r^2 ^values ranged from 0.001 to 0.367 and were all significant (p < 0.05) except for the pair consisting of SNPs c.4500A>C and SNPc.4950C>T.

**Table 1:** Genotypic and allelic frequencies of CARD15 SNPs.

	**Genotypes**		**Alleles**	
	**No**.	**Frequency (%)**	**No**.	**Frequency (%)**
**c.2886-14A>G**				

**AA (A)**	228	67.5	556	82.2
**AG**	100	29.5		
**GG (G)**	10	3.0	120	17.8

**c.3020A>T**				

**AA (A)**	93	27.5	365	54.0
**AT**	179	53.0		
**TT (T)**	66	19.5	311	46.0

**c.4500A>C**				

**AA (A)**	42	12.4	254	37.6
**AC**	170	50.3		
**CC (C)**	126	37.3	422	62.4

**c.4950C>T**				

**TT (T)**	250	74.0	580	85.8
**TC**	80	23.6		
**CC (C)**	8	2.4	96	14.2

### SNP association analyses

Statistical analyses revealed associations between *CARD15 *SNP c.3020A>T and EBVs for milk yield, protein yield, udder depth, and SCS and between SNP c.4500A>C and milk yield, fat yield and protein yield. Amongst all the possible regression models for SCS, a single SNP model including SNP c.3020A>T was found to be the best model and showed significant association of this SNP with SCS (p = 0.0097). The average allele substitution effect of this SNP for SCS was 0.068, with allele 'T' increasing SCS over allele 'A' (21% of the SD for SCS EBV). All significant associations were retained at 8% experimental-wise significance level by permutation test. A complete description of the average allele substitution effects is presented in Table 2.

**Table 2:** Average allele substitution effect of SNPs c.3020A>T and c.4500A>C on EBVs for milk yield (Milk), fat yield (Fat), protein yield (Prot), udder depth (UD) and somatic cell score (SCS).

	**Trait**				
	
	**Milk (Kg)**	**Fat (Kg)**	**Prot (Kg)**	**UD**	**SCS**
**SNP**	**Allele substitution effects ± SE (comparison-wise P level)**				

***c.3020A>T***	359.77 ± 107.70 (<0.0001^++^)	-	9.90 ± 3.20 (0.0003^++^)	-1.10 ± 0.41 (0.0087^+^)	0.07 ± 0.03 (0.0097^+^)
***c.4500A>C***	348.44 ± 117.80 (<0.0001^++^)	8.29 ± 2.40 (0.0010^++^)	11.31 ± 3.51 (<0.0001^++^)	-	-
**Power**^1^	0.93	0.74	0.93	0.56	0.54

^++^Significant effect by permutation test at an experimental-wise significance level of 5%

^+^Significant effect by permutation test at an experimental-wise significance level of 8%

^1 ^Power of the analysis considering an experimental-wise significance level of either 5% (for Milk, Fat and Prot) or 8% (for UD and SCS).

### Haplotype analysis

Two *CARD15 *SNPs (c.3020A>T and c.4500A>C) were used for haplotype reconstruction. The estimated haplotype frequencies were 42.6%, 26.1%, 19.8% and 11.4% for Hap12(AC), Hap21(TA), Hap22(TC) and Hap11(AA), respectively. The linear effects of each of the haplotypes were estimated by treating the effect of the most frequent haplotype (Hap12) as a control and contrasting the effects of the other haplotypes (Hap21, Hap22 and Hap11) against it (Table 3). Analysis revealed statistically significant differences between Hap21 and Hap12 for SCS and fat yield, and between Hap22 and Hap12 for milk yield and protein yield at different levels of significance. All significant associations were retained at 8% experimental-wise level by permutation test.

**Table 3:** Linear effects of *CARD15 *on EBVs for milk yield (Milk), fat yield (Fat), protein yield (Prot), udder depth (UD) and somatic cell score (SCS), when comparing haplotypes Hap11, Hap22, and Hap21, against Hap12.

	**Trait**				
	
	**Milk (Kg)**	**Fat (Kg)**	**Prot (Kg)**	**UD**	**SCS**
**Haplotypes**	**Haplotype effects ± SE (P level)**				

***Hap11(AA)***	-325.2 ± 175.30	-8.23 ± 5.60	-12.68 ± 5.20	-0.91 ± 1.00	0.02 ± 0.06
	(0.06)	(0.14)	(0.01)	(0.36)	(0.74)
***Hap22(TC)***	523.5 ± 129.70	6.89 ± 4.15	13.15 ± 3.85	-1.71 ± 0.74	0.05 ± 0.05
	(<0.0001^++^)	(0.07)	(0.0007^++^)	(0.02)	(0.26)
***Hap21(TA)***	-57.5 ± 81.62	-7.27 ± 2.61	-3.10 ± 2.42	-0.93 ± 0.46	0.07 ± 0.03
	(0.48)	(0.0057^++^)	(0.20)	(0.04)	(0.012^+^)
**Power**^1^	0.91	0.69	0.90	0.49	0.38

^++^Significant effect by permutation test at an experimental-wise significance level of 5%

^+^Significant effect by permutation test at an experimental-wise significance level of 8%

^1^Power of the analysis considering an experimental-wise significance level of either 5% (for Milk, Fat and Prot) or 8% (for UD and SCS).

## Discussion

TLR2 and CARD15 play a role in the initiation of inflammatory and immune responses to bacterial infections. A number of studies have reported that SNPs in PRMs of different species play an important role in contributing towards disease susceptibility. Therefore, the main objective of this study was to identify SNPs in the *TLR2 *and *CARD15 *and to estimate the extent of associations between these SNPs and SCS, a trait directly related to udder inflammation. In the current experiment, the DNA pool used for the purpose of SNP detection was comprised of DNA samples from 40 animals with extreme EBVs for SCS. The exons, flanking intronic sequences, and promoter region were targeted for SNP detection. Therefore it is unlikely, that all SNPs in the population were identified.

Although no SNPs were identified in *TLR2*, four SNPs were identified in *CARD15*; only one SNP was found in the coding region (c.3020A>T) of this gene. This SNP, located in exon 12, is a non-synonymous SNP coding the terminal LRR domain of CARD15 receptor. While allele 'A' at this position codes for glutamine, allele 'T' codes for leucine. The association of the T allele with increased SCS and decreased udder depth, which predisposes animals to mammary infections, indicates that changes in the composition of the LRR domain of CARD15 may contribute to disease susceptibility. The terminal LRR domains, as in CARD15, are common to different PRMs and are responsible for PAMP recognition. There are several reports of polymorphisms in the human and mouse LRR coding gene segments that contribute to differential binding to several bacterial components [15,24,27]. In humans, *CARD15 *variants have been hypothesized to alter bacterial component recognition by altering the structure of LRR domain or the adjacent region [23]. It is possible that SNP c.3020A>T may compromise protein functionality by altering the conformation of the binding site in a similar fashion. This could contribute to the development of inflammatory disorders and warrants further investigation. The association between this SNP and udder depth might be an indirect result of the genetic correlation that exists between SCS and udder depth (rg= -0.26) [28].

Strong associations were also observed between the analyzed *CARD15 *SNPs and production traits. Such associations may be a result of linkage between these SNPs and other genes on the same chromosome having a significant effect on these production traits. Significant QTLs have been found on Bta18 for all the traits used in the analysis. While QTLs for SCS and udder composite index exist close to the location of this gene (about 230 kbs apart)[29,30] on the chromosome, QTLs for milk yield, protein yield and fat yield were situated farther away (>1,500 kbs apart) [30-32].

Haplotype reconstruction revealed all four possible *CARD15 *haplotypes to be segregating in the sampled bulls. The second most frequent haplotype (Hap21) differed significantly from the most common haplotype (Hap12) with respect to its effect on SCS EBVs. In agreement with the results of the allele substitution analyses, the linear effect of Hap21 carrying the allele 'T' at SNP position c.3020A>T was significantly different from Hap12. However, we did not see any difference between the linear effects of Hap22 and Hap12 for SCS. Strong associations were observed between Hap22 and milk and protein yields (p < 0.0001 and p = 0.0007 respectively). Since, Hap22 carries alleles 'T' and 'C', both of which are associated with increased milk and protein yields, these results were in agreement with the allele substitution analysis. It is interesting to note that the most common haplotype, Hap12, carries alleles 'A' and 'C' at positions c.3020A>T and c.4500A>C respectively, and is beneficial not only for reducing SCS but also for increasing production. Thus selection for Hap12 in the population seems promising.

## Conclusion

In conclusion, four SNPs in *CARD15 *in a sample of Canadian Holstein bulls were discovered. Statistical analyses revealed that SNP c.3020A>T was associated with EBVs for SCS and udder depth and milk and protein yields, while SNP c.4500A>C was only associated with milk, fat and protein yields. The most common haplotypes for these two SNPs in the population differed significantly for their effect on SCS. Moreover, the most common haplotype carried alleles at both loci that are favourable for reducing SCS and increasing production EBVs. This implies that these two SNP, together with other gene polymorphisms, may be potentially used for genetic selection for mastitis resistance and production. The findings of this study indicate that SNP c.3020A>T is a candidate for further detailed studies on its functional mechanisms.

## Methods

### Resource population

The resource population consisted of 2166 Holstein bulls selected on the basis of extreme EBV for either protein yield or SCS. A total of 338 semen samples of these bulls were selected within families on the basis of extreme EBVs for either protein yield or SCS for this study. The EBV's for SCS, udder depth, milk yield, protein yield and fat yield of these bulls were obtained from a national genetic evaluation database generated in August 2006 by the Canadian Dairy Network [33], Guelph, Ontario, Canada, and used in the association studies. Table 4 provides descriptive statistics of the EBVs of the bulls. The majority of the selected population consisted of half sib families from 20 sires, the size of which ranged from 2 to 30 bulls. Semen samples were kindly provided by Semex Alliance (Guelph, Ontario, Canada).

**Table 4:** Descriptive statistics of the EBV of the Holstein bulls sampled for the association study.

	EBV				
	
	Milk yield (kg)	Fat yield (kg)	Protein yield (kg)	Udder depth	SCS^1 ^(score)
N^2^	337	337	337	335	335
Mean	115.59	4.83	5.65	0.46	3.01
SD	935.95	29.63	27.75	5.24	0.33
Max	2173	101	63	12	4.21
Min	-2358	-81	-76	-12	2.34

^1 ^SCS = Somatic cell score [log(2) scores]

^2 ^Number of records. One bull had missing EBV for Milk, Fat and Protein yield; and three bulls had missing EBV for Udder depth and SCS.

### DNA extraction

A slightly modified standard phenol chloroform procedure was used to extract DNA from semen samples [34]. An Eppendorf Biophotometer (Berlin, Germany) was used to assess the DNA concentration and quality on the basis of absorbance of UV light at 260 (A_260_) and 280 nm (A_280_).

### Construction of DNA Pools for SNP Detection

Twenty bulls with high and twenty bulls with low EBVs for SCS were selected from the previously selected 338 animals, to create a DNA pool containing equal amounts of DNA from each bull. The descriptive statistics of the EBVs of the bulls used for DNA pooling are given in Table 5. Individual DNA samples were quantified and diluted using the PicoGreen dsDNA quantification procedure (Molecular Probes; Invitrogen, Carlsbad, CA) on a Victor 3 flourescent plate reader (Perkin Elmer, Wellesley, MA), until the concentration of all 40 DNA samples was 8 ± 1 ng/μl. Equal volumes of DNA from each of the 40 samples were aliquoted to a common tube to construct the DNA pool. This pool was used in PCR reactions to amplify the *CARD15 *exons and its flanking introns and a 2 kb promoter region, and the *TLR2 *coding exon, flanking introns and a 2 kb promoter region. The primers used for SNP discovery in *TLR2 *and *CARD15 *are shown in Table 6 and Table 7 respectively. Each PCR amplicon was sequenced in forward and reverse directions for SNP discovery. Polymorphisms were detected by scrutinizing the forward and reverse electropherograms generated from the sequencer.

**Table 5:** Descriptive statistics of the EBV of the Holstein bulls used for DNA pooling.

	EBV				
	
	Milk yield (kg)	Fat yield (kg)	Protein yield (kg)	Udder depth	SCS^1 ^(score)
N^2^	40	40	40	39	39
Mean	19.95	-1.4	2.15	0.18	3.04
SD	853.33	31.48	25.00	5.50	0.46
Max	1714	56	47	12	3.88
Min	-2246	-67	-60	-9	2.34

^1^SCS= Somatic cell score [log(2) scores]

^2 ^Number of records. One bull had missing EBV for Udder depth and SCS.

**Table 6:** PCR primers and conditions for identification of SNPs in *TLR2*.

**Exon**	**Primer name**	**Sequence, 5' → 3'**	**T**_**ann**_, **°C**	**Amplicon size, bp**
***TLR2 *PROMOTER**				

**Part 1**	Forward	CCCTTGAATGTATTTGTCACTTCC	60	664
	Reverse	TCCATATTTTTGAGAATCTGACTGA		
**Part 2**	Forward	TGCAAGGATTACAGCAGATTTTC	59	579
	Reverse	AAAATCATGTATTTACCCACAACG		
**Part 3**	Forward	CGTACAAAGATGTGCCAGAGG	59	586
	Reverse	CCAGTGTTCTTGCCTGGAGA		
**Part 4**	Forward	GGGGCCTTTAATGAGACCTG	60	445
	Reverse	GCCCTTCCTAAATTGAGATCAGA		
**Part 5**	Forward	GTGCCTTTGCAATGAGGACT	60	496
	Reverse	GCCTGAAGAGCAGATGAGGA		

***TLR2 *GENE**				

**Part 1**	Forward	GCCATGATGTCAAACACAGTCA	58	613
	Reverse	AGCACTGATCTCAAGCTCCTCA		
**Part 2**	Forward	CAAAACACTTGGGGAAACATCT	59	611
	Reverse	TTTTAACTCTGCCTGTGAGTGGA		
**Part 3**	Forward	ACTGTACCCATGATGGAATTGG	61	643
	Reverse	ACGGGTAAGAAGGAGGCATCT		
**Part 4**	Forward	CACAGTTTAACCCAGTGCCTTC	59	633
	Reverse	CATGAGGTTCTCCACCCAGTAG		
**Part 5**	Forward	CGGACTGTGGTACATGAAGATG	60	643
	Reverse	TCAGCATCAGTTCTTCCAATGA		

**Table 7:** PCR primers and conditions for identification of *CARD15 *single-nucleotide polymorphisms.

**Exon**	**Primer name**	**Sequence, 5' → 3'**	**T**_**ann**_, **°C**	**Amplicon size, bp**
***CARD15 *PROMOTER**				

**Part 1**	Forward	CCCCACTTCTATCCTCTGGACT	58	640
	Reverse	AGCAGAGGAAATCTCTCAGCAG		
**Part 2**	Forward	GACTTGGCCATGCCTACTCTAA	59	601
	Reverse	GAAACTGAGGCAGCCAGGTAG		
**Part 3**	Forward	TAGACACAGAGTGCTGGCAAGA	58	625
	Reverse	CCCGCAAAGATCATTAGGTTTA		
**Part 4**	Forward	TCTTTCCTATCTGCAGCTGTCC	59	632
	Reverse	AAGTCCCATCTCTGTCCTAGCC		

***CARD15 *GENE**				

**1**	Forward	TGTAGACAGACGCTGGAGTTCCTCT	62	322
	Reverse	ATGCACTGACTCCCATCTACTGCTC		
**2**	Forward	GAGAAGCCCTGCCCTGACCT	63	599
	Reverse	ACTATGACCCACATCTCCCCACAG		
**3**	Forward	TGCATCTTACCATGCAGATGTTTTTC	62	392
	Reverse	GAGACGCTGAGTTTTACGGAGGTTT		
**4 (part1)**	Forward	CTTGTTAGTGGAGAGCCAGGAC	58	546
	Reverse	AACAGCAGTGTTCGAAGAGCA		
**4 (part2)**	Forward	TTCTCTTCGTCTTCCCATTTAGC	59	506
	Reverse	GGACACCATCCAGGAGAAGAC		
**4 (part3)**	Forward	CATCGAACTGTACCTGAGGAAGC	60	594
	Reverse	AGGAACAATTTGGGCAGCAC		
**4 (part4)**	Forward	TCAGACATCTCTTCCAAGATCACA	59	680
	Reverse	ACTCCTGGCTCCCAGCATAA		
**56**	Forward	GGGTTAAGCAAAAGTCTCTGTGG	59	595
	Reverse	AATGAGACACTGTCCTTGTTTTCAG		
**7**	Forward	CAGGTCTTGGGAGCAGTAAGG	59	375
	Reverse	ACATCCTGAGCTTCCTGTTTATTG		
**8**	Forward	CTCACTTGCTGGGACCTGAGT	59	285
	Reverse	TCCCTCCTCACACTGGCTTC		
**9**	Forward	AACGATTAGTCTGAAATGGAGCAG	58	300
	Reverse	TACACACACATCAGCTTCCACAG		
**10**	Forward	CCTTACACTTTGCTTGACCTGTTT	58	472
	Reverse	ACCCCGAAAGATTGTTTTCTAGG		
**11**	Forward	GAATTCATTGGGAATCTCAGACAG	59	355
	Reverse	CAGGACTAGAGGTCTGAGCCATAA		
**12(part1)**	Forward	ACAGGTTTACAAAGCAGCATCTTC	59	578
	Reverse	ACCACTCAACCTGATGGATGAC		
**12(part2)**	Forward	GGCTGGTCTCAAGTCAAGCTG	60	619
	Reverse	GCACAAACAAAACCAAACCATC		
**12(part3)**	Forward	GAACAACTCTGTATCCAAATGCAAC	59	641
	Reverse	ACGCTGGGCTAGACTTCTCTAAAC		
**12(part4)**	Forward	TCTCCTTGAAGGGAGGAGACAT	59	509
	Reverse	CACAACGCTGTAAATCAACCATAC		
**12(part5)**	Forward	GGCCTTGGTGAAATAATTCTTAGC	58	588
	Reverse	CAGCAACATGGATGGATCTAGAAA		

### SNP Genotyping

**Figure 2:**
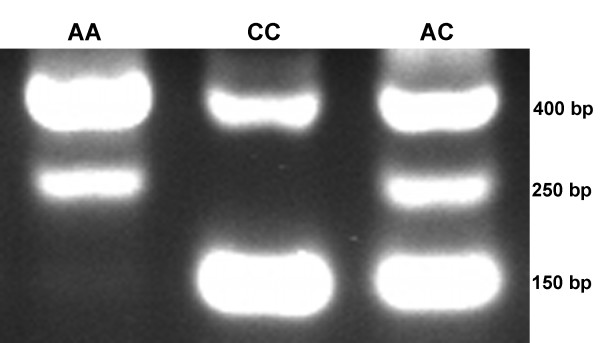
**Agarose gel differentiating three genotypes**. Agarose gel (2.5%) stained with ethidium bromide showing tetra-primer ARMS-PCR amplicons differentiating the three *CARD15 *genotypes for SNP c.4500A>C.

The tetra-primer Amplification Refractory Mutation System (ARMS)-PCR procedure, as described by Ye et al. [35], was used to genotype all *CARD15 *SNPs. This method of genotyping is simple and economical, involving a single PCR resaction followed by gel electrophoresis. Primers were designed using the online primer design facility made accessible by Ye et al. [36]. The primer sets used for genotyping each identified SNP are shown in Table 8. The PCR reactions were performed in a total volume of 10 μl, containing 10 pmol of each of the inner primers, 1 pmol of each of the outer primers, 200 μM deoxyribonucleotide triphosphates, 2.5 mM MgCl_2_, 1× PCR buffer, and 0.5 units of AmpliTaq Gold DNA polymerase (Applied Biosystems, Foster City, CA). To increase the specificity of the reaction, a touchdown profile was followed. For touchdown reactions the annealing temperature was 4°C higher for the first cycle, decreasing by 1°C per cycle until the annealing temperature indicated in Table 8 was reached, then continuing at that temperature in the annealing step of the remaining cycles. The PCR profile was as follows: 95°C for 8 min, 34 cycles of 30 s at 94°C, 34 cycles of 30s at annealing temperature (including initial touchdown cycles), and 30s of extension at 72°C, ending with 5 min at 72°C. The annealing temperatures are shown in Table 8. A T-GRADIENT thermocycler (Biometra, Montreal Biotech Inc. Kirkland, Canada) was used to carry out the reactions. A 8 μl aliquot of the PCR products was mixed with 2 μl of loading buffer and subjected to horizontal agarose gel (2.5%) electrophoresis. The gels were stained with ethidium bromide for visualization and the genotypes were determined as shown in Figure 2.

**Table 8:** The tetra-primer Amplification Refractory Mutation System-PCR primers and conditions for genotyping *CARD15 *SNPs.

**Position**	**Primer Name**	**Sequence, 5' → 3'**	**T**_**ann**_, **°C**	**Amplicon size, bp**
**c.2886-14A>G**	Forward inner primer Reverse inner primer Forward outer primer Reverse outer primer	CAAGCATCCTCAAAGTTCACCATTATG GTGGTTGTTAGACAGCCTAGAGAGGAATAT CCTGGCACTAAAATGCACGTATTTTTAT AGTTATACGTTGGAAAACTGAGGCTCAG	60°C	405bp(outer) 300 bp (allele 'A') 150 bp (allele 'G')
**c.3020A>T**	Forward inner primer Reverse inner primer Forward outer primer Reverse outer primer	GAGGAAATTGAGAAACTCAGCCAGCA AAACATCAGAGCAAGAGTCTGGTATGCA CTGACAGAGGGTAAAACCTGCACAGACG CTGTGCCAGAACAAAGGTGACCTATTG	59°C	310bp (outer) 197 bp (allele 'A') 166 bp (allele 'T')
**c.4500A>C**	Forward inner primer Reverse inner primer Forward outer primer Reverse outer primer	AGAGACGCAAGCAGGCCCCTGGGCCGCA CCCTGGAGACACTTGGAGAGAATGGGGG AGCAGTGTTTAGAAATAGCCTCGCAAT AGAGAACCCACACACATGCCCTTACTG	64°C	400bp (outer) 250 bp (allele 'A') 150 bp (allele 'C')
**c.4950C>T**	Forward inner primer Reverse inner primer Forward outer primer Reverse outer primer	TGTATCCATTCAATATATAATACATTGGC GGCTAGCAAATGTATTATGAGGTGAA TTCATTTGTTTGAATTTATTTTATTGAGG GAATGTAACACAAAGGAACATATCTACAA	59°C	341bp (outer) 226 bp (allele 'T') 169 bp (allele 'C')

### Statistical Analyses

#### Average allele substitution effects

Preliminary analyses revealed only two *CARD15 *SNPs (c.3020A>T and c.4500A>C) to be associated with any of the different traits analyzed; only these two SNPs were used for the detailed analysis. Data was analyzed using PROC REG (SAS Institute, Inc., Cary, NC), by the following model:

yj=μ+∑i=12βiGi+ej
 MathType@MTEF@5@5@+=feaafiart1ev1aaatCvAUfKttLearuWrP9MDH5MBPbIqV92AaeXatLxBI9gBaebbnrfifHhDYfgasaacPC6xNi=xI8qiVKYPFjYdHaVhbbf9v8qqaqFr0xc9vqFj0dXdbba91qpepeI8k8fiI+fsY=rqGqVepae9pg0db9vqaiVgFr0xfr=xfr=xc9adbaqaaeGacaGaaiaabeqaaeqabiWaaaGcbaGaemyEaK3aaSbaaSqaaiabdQgaQbqabaGccqGH9aqpiiGacqWF8oqBcqGHRaWkdaaeWbqaaiab=j7aInaaBaaaleaacqWGPbqAaeqaaOGaem4raC0aaSbaaSqaaiabdMgaPbqabaGccqGHRaWkcqWGLbqzdaWgaaWcbaGaemOAaOgabeaaaeaacqWGPbqAcqGH9aqpcqaIXaqmaeaacqaIYaGma0GaeyyeIuoaaaa@42E1@

where: *y*_*j *_= Trait EBV for the jth animal; *μ *= Overall mean, *β*_*i *_= regression coefficient (allele substitution effect) for the ith SNP, *e*_*j *_= random error and *G*_*i *_= the genotype of the ith SNP recoded as in Zeng et al [37]:

Gi=1 for a homozygote (eg.,AA)0 for a Heterozygote (eg.,AT)−1 for a homozygote (eg.,TT)
 MathType@MTEF@5@5@+=feaafiart1ev1aaatCvAUfKttLearuWrP9MDH5MBPbIqV92AaeXatLxBI9gBaebbnrfifHhDYfgasaacPC6xNi=xI8qiVKYPFjYdHaVhbbf9v8qqaqFr0xc9vqFj0dXdbba91qpepeI8k8fiI+fsY=rqGqVepae9pg0db9vqaiVgFr0xfr=xfr=xc9adbaqaaeGacaGaaiaabeqaaeqabiWaaaGcbaGaem4raC0aaSbaaSqaaiabdMgaPbqabaGccqGH9aqpfaqaaeWabaaabaGaeGymaeJaeeiiaaIaeeOzayMaee4Ba8MaeeOCaiNaeeiiaaIaeeyyaeMaeeiiaaIaeeiAaGMaee4Ba8MaeeyBa0Maee4Ba8MaeeOEaONaeeyEaKNaee4zaCMaee4Ba8MaeeiDaqNaeeyzauMaeeiiaaIaeiikaGIaeeyzauMaee4zaCMaeiOla4IaeiilaWIaeeyqaeKaeeyqaeKaeiykaKcabaGaeGimaaJaeeiiaaIaeeOzayMaee4Ba8MaeeOCaiNaeeiiaaIaeeyyaeMaeeiiaaIaeeisaGKaeeyzauMaeeiDaqNaeeyzauMaeeOCaiNaee4Ba8MaeeOEaONaeeyEaKNaee4zaCMaee4Ba8MaeeiDaqNaeeyzauMaeeiiaaIaeiikaGIaeeyzauMaee4zaCMaeiOla4IaeiilaWIaeeyqaeKaeeivaqLaeiykaKcabaGaeyOeI0IaeGymaeJaeeiiaaIaeeOzayMaee4Ba8MaeeOCaiNaeeiiaaIaeeyyaeMaeeiiaaIaeeiAaGMaee4Ba8MaeeyBa0Maee4Ba8MaeeOEaONaeeyEaKNaee4zaCMaee4Ba8MaeeiDaqNaeeyzauMaeeiiaaIaeiikaGIaeeyzauMaee4zaCMaeiOla4IaeiilaWIaeeivaqLaeeivaqLaeiykaKcaaaaa@9272@

The recoded genotypes are listed in Table 1. Mallow's criterion was used to select the final regression model. Type I experimental errors were controlled by implementing permutation test [38]. The traits of interest were milk yield, fat yield, protein yield, udder depth, and somatic cell score. For each trait, the power of the analysis was calculated using a software developed and described by Dunlap et al. [39], which takes into account the significance level used, number of independent variables, sample size, and the coefficient of determination of the multiple regression model.

### Genotype Frequencies

The genotypic frequencies were tested for deviations from proportions of Hardy Weinberg Equilibrium. This was performed applying a Chi-square test. The pair-wise level of linkage disequilibrium was measured using the squared correlation of the alleles at two loci (r^2^) [40] and was tested for significance using a Chi-square test [41]:

xdf=12=(n−3)z2,
 MathType@MTEF@5@5@+=feaafiart1ev1aaatCvAUfKttLearuWrP9MDH5MBPbIqV92AaeXatLxBI9gBaebbnrfifHhDYfgasaacPC6xNi=xI8qiVKYPFjYdHaVhbbf9v8qqaqFr0xc9vqFj0dXdbba91qpepeI8k8fiI+fsY=rqGqVepae9pg0db9vqaiVgFr0xfr=xfr=xc9adbaqaaeGacaGaaiaabeqaaeqabiWaaaGcbaGaemiEaG3aa0baaSqaaiabdsgaKjabdAgaMjabg2da9iabigdaXaqaaiabikdaYaaakiabg2da9iabcIcaOiabd6gaUjabgkHiTiabiodaZiabcMcaPiabdQha6naaCaaaleqabaGaeGOmaidaaOGaeiilaWcaaa@3CE3@

where, *n *is the number of bulls genotyped and

z=0.5ln⁡(1+r1−r).
 MathType@MTEF@5@5@+=feaafiart1ev1aaatCvAUfKttLearuWrP9MDH5MBPbIqV92AaeXatLxBI9gBaebbnrfifHhDYfgasaacPC6xNi=xI8qiVKYPFjYdHaVhbbf9v8qqaqFr0xc9vqFj0dXdbba91qpepeI8k8fiI+fsY=rqGqVepae9pg0db9vqaiVgFr0xfr=xfr=xc9adbaqaaeGacaGaaiaabeqaaeqabiWaaaGcbaGaemOEaONaeyypa0JaeGimaaJaeiOla4IaeGynauJagiiBaWMaeiOBa4MaeiikaGscfa4aaSaaaeaacqaIXaqmcqGHRaWkcqWGYbGCaeaacqaIXaqmcqGHsislcqWGYbGCaaGccqGGPaqkcqGGUaGlaaa@3DFB@

### Haplotype Construction and Analyses

The haplotype probabilities were reconstructed using the program 'HAPROB' developed by Boettcher et al. [42]. This program is based on an algorithm that uses a two-step Monte Carlo-based approach to estimate haplotype probabilities for the genotyped members of half-sib families where the parents lack genotypic information. The first step estimated the haplotype probabilities for the sires based on the offspring genotypes and population allelic frequencies while the second step estimated the offspring-haplotype probabilities based on the sire probabilities and population frequencies. These two steps were alternately iterated until the estimated population frequencies converged to stable values. The final results were a set of estimated haplotype probabilities for each animal.

Haplotype effects were estimated by regressing EBV's on haplotype probabilities, which are expressed as the expected number of copies of each haplotype. The following model was used for statistical analyses using SAS software (SAS Institute, 1999):

yj=μ+∑i=14βiHapij+ej
 MathType@MTEF@5@5@+=feaafiart1ev1aaatCvAUfKttLearuWrP9MDH5MBPbIqV92AaeXatLxBI9gBaebbnrfifHhDYfgasaacPC6xNi=xI8qiVKYPFjYdHaVhbbf9v8qqaqFr0xc9vqFj0dXdbba91qpepeI8k8fiI+fsY=rqGqVepae9pg0db9vqaiVgFr0xfr=xfr=xc9adbaqaaeGacaGaaiaabeqaaeqabiWaaaGcbaGaemyEaK3aaSbaaSqaaiabdQgaQbqabaGccqGH9aqpiiGacqWF8oqBcqGHRaWkdaaeWbqaaiab=j7aInaaBaaaleaacqWGPbqAaeqaaOGaemisaGKaemyyaeMaemiCaa3aaSbaaSqaaiabdMgaPjabdQgaQbqabaGccqGHRaWkcqWGLbqzdaWgaaWcbaGaemOAaOgabeaaaeaacqWGPbqAcqGH9aqpcqaIXaqmaeaacqaI0aana0GaeyyeIuoaaaa@46F8@

where y_*j *_is trait EBV of the jth animal, β_*i *_is the linear regression coefficient for the *i*th haplotype, Hap_ij _is the probability of the ith haplotype for the jth bull, *e*_*j *_are random residual effects. Permutation test was implemented to control type I experimental-wise errors. For each trait, the power of the haplotype analysis was calculated using a software developed and described by Dunlap et al. [39].

## Authors' contributions

SDP participated in the design of the study, carried out DNA pooling, sequencing of pooled DNA for SNP detection, SNP genotyping, haplotype reconstruction, performed the SNP analyses, haplotype analyses and drafted the manuscript. FSS participated in haplotype reconstruction, designed the SNP and haplotype analyses, and helped to draft the manuscript. IL helped with DNA pooling, SNP detection and genotyping. BSS performed the DNA extractions from semen samples used in this study and participated in design of the study and statistical analyses. NAK designed and supervised the study, participated in its coordination, provided necessary funding and resources and revised the manuscript. All authors read and approved the final manuscript.
